# Anti-Inflammatory and Anti-Fibrotic Effect of Immortalized Mesenchymal-Stem-Cell-Derived Conditioned Medium on Human Lung Myofibroblasts and Epithelial Cells

**DOI:** 10.3390/ijms23094570

**Published:** 2022-04-20

**Authors:** Eirini Filidou, Leonidas Kandilogiannakis, Gesthimani Tarapatzi, Michail Spathakis, Paschalis Steiropoulos, Dimitrios Mikroulis, Konstantinos Arvanitidis, Vasilis Paspaliaris, George Kolios

**Affiliations:** 1 Laboratory of Pharmacology, Faculty of Medicine, Democritus University of Thrace, 68100 Alexandroupolis, Greece; efilidou@hotmail.com (E.F.); lkandilo@med.duth.gr (L.K.); mtarapagi@gmail.com (G.T.); michael.spathakis@outlook.com (M.S.); karvanit@med.duth.gr (K.A.); gkolios@med.duth.gr (G.K.); 2 Department of Pneumonology, Medical School, Democritus University of Thrace, 68100 Alexandroupolis, Greece; pstirop@med.duth.gr; 3 Department of Cardiac Surgery, Democritus University of Thrace, University Hospital of Alexandroupolis, 68100 Alexandroupolis, Greece; dmikrou@med.duth.gr; 4 Vasilis Paspaliaris, Tithon Biotech Inc., 11440 West Bernardo Court, Suite 300, San Diego, CA 92127, USA

**Keywords:** ADSCs, mesenchymal stem cells, conditioned medium, pulmonary subepithelial myofibroblasts, A549, idiopathic pulmonary fibrosis, fibrosis, inflammation

## Abstract

Idiopathic pulmonary fibrosis (IPF) is caused by progressive lung tissue impairment due to extended chronic fibrosis, and it has no known effective treatment. The use of conditioned media (CM) from an immortalized human adipose mesenchymal stem cell line could be a promising therapeutic strategy, as it can reduce both fibrotic and inflammatory responses. We aimed to investigate the anti-inflammatory and anti-fibrotic effect of CM on human pulmonary subepithelial myofibroblasts (hPSM) and on A549 pulmonary epithelial cells, treated with pro-inflammatory or pro-fibrotic mediators. CM inhibited the proinflammatory cytokine-induced mRNA and protein production of various chemokines in both hPSMs and A549 cells. It also downregulated the mRNA expression of IL-1α, but upregulated IL-1β and IL-6 mRNA production in both cell types. CM downregulated the pro-fibrotic-induced mRNA expression of collagen Type III and the migration rate of hPSMs, but upregulated fibronectin mRNA production and the total protein collagen secretion. CM’s direct effect on the chemotaxis and cell recruitment of immune-associated cells, and its indirect effect on fibrosis through the significant decrease in the migration capacity of hPSMs, makes it a plausible candidate for further development towards a therapeutic treatment for IPF.

## 1. Introduction

Idiopathic pulmonary fibrosis (IPF) is the most common interstitial lung disease (ILD), affecting 3 million people worldwide [1]. It is highly heterogenous among patients and can manifest in various ways, often leading to misdiagnoses and delayed treatment [2,3]. IPF is caused by progressive lung tissue impairment due to extended chronic fibrosis of unknown etiology, and ultimately leads to death [4]. Current in silico approaches may provide new information regarding the development of IPF, for instance, establishing early disease diagnosis, disease severity and assessing treatment efficacy [5,6,7,8].

Unfortunately, our current knowledge is unable to provide a cure for IPF, and even symptomatic treatment can be difficult [9], especially with the acute exacerbation of IPF [10,11]. Recent developments in anti-fibrotic agents, such as Nintedanib and Pirfenidone, were shown to slow the progression of IPF when used as monotherapies or possibly as combined therapies [12,13,14] with more agents, such as BG00011, BMS-986020, CC-90001, Co-trimoxazole, GLPG1690, Lebrikizumab, Omipalisib, Pamrevlumab (FG-3019), PBI-4050, Pentraxin-2, Rituximab, Sildenafil, Simtuzumab, STX-100, Tipelukast, Tralokinumab, which are being thoroughly studied [15,16].

Although both Nintedanib and Pirfenidone are the only two approved anti-fibrotic drugs that were shown to improve the forced vital capacity and the overall life quality of patients for a noticeably extended period of time, neither of these two drugs have been able to stall or reverse the overall disease progression or change the high mortality that remains 3–5 years after the initial diagnosis [17]. Therefore, it is crucial that novel pharmacological approaches are developed for the successful treatment of IPF. While stem cell transplantation is a possible new therapeutic method under clinical investigation [18,19], it has raised a wide variety of ethical as well as technical concerns [20,21,22]. Furthermore, the microenvironment of a pathological organ could make live stem cells prone to worsening the situation, eventually causing more harm to than help for the patients [23,24]. Due to these problems, the alternative therapies that are currently being developed include cell-culture conditioned media (CM) and their derived extracellular vesicles (EVs) [25,26].

Using human bronchial epithelial-cell-derived or fibroblast-derived or even bone marrow mesenchymal-stem-cell-derived EVs containing miRNAs targeting key fibrotic cascades instead of intact cells appears more promising than stem cell transplantation [27,28,29], as do CM from stem cells, which proved to be able to reduce both fibrotic and inflammatory responses in a wide variety of pathologies [30,31,32,33,34,35]. More specifically, CM used for in vivo and in vitro models show great potential in reducing pro-inflammatory signals [30,36,37,38,39,40,41], augmenting anti-inflammatory pathways [40], decreasing the fibrotic process [36,37,42,43,44] and promoting tissue regeneration [30,39,45,46,47], which all contribute to slowing IPF progression. Towards this notion, we recently showed that CM from immortalized human adipose-derived stem cells (ADSCs) had anti-fibrotic and anti-inflammatory effects in a bleomycin-induced fibrosis mouse model, ultimately reversing the established fibrosis in mice [48]. However, CM need further standardization as to the therapeutic dosages and frequency of administration required for IPF [49].

Myofibroblasts are key mediators of IPF fibrosis, not only by producing extracellular matrix proteins but also through various other factors included in their secretome [50,51,52,53]. It was previously shown by Kheirollahi et al. that therapies targeting fibrotic pathways in myofibroblasts could ameliorate IPF progression [38], and modifying those cascades as well as reversing the fibroblast differentiation to myofibroblasts could also create potential therapeutic targets for IPF [54,55,56,57,58,59]. In our study, we investigate the anti-inflammatory and anti-fibrotic effect of CM-ADSCs on human pulmonary subepithelial myofibroblasts (hPSM) and on the A549 pulmonary epithelial cells, which are under the influence of pro-inflammatory or pro-fibrotic mediators.

## 2. Results

### 2.1. The Conditioned Medium Inhibits the IL-1α- and TNF-α-Induced mRNA and Protein Chemokine Production in the Pulmonary Epithelial Cell Line A549 and in hPSMs

In order to investigate the possible anti-inflammatory properties of the CM, we first examined its effect on the IL-1α- and TNF-α-induced mRNA and protein production of the chemokines, CCL2, CCL20, CXCL1, CXCL8, CXCL10 and CXCL11, in the pulmonary epithelial cell line A549 and in hPSMs, as it is well known that these chemokines play a major role in the chemotaxis of immune-associated cells [60] and are inducible by IL-1α and TNF-α [61,62].

**Figure 1:**
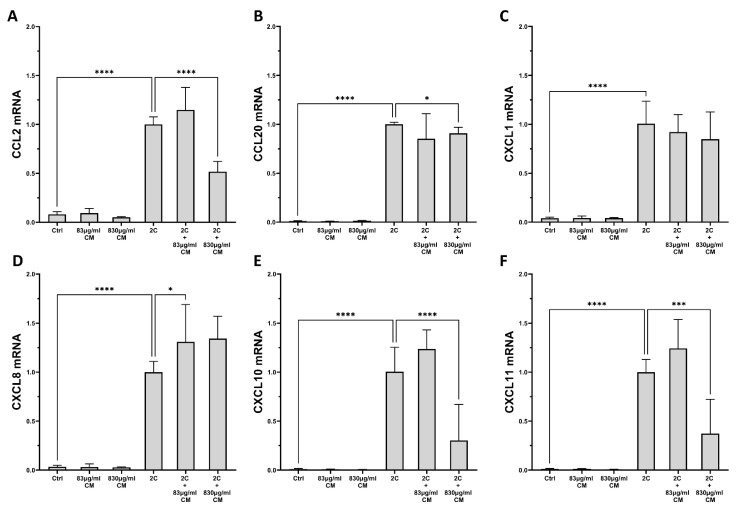
The effect of CM on chemokine mRNA expression in A549 pulmonary epithelial cells, which are under pro-inflammatory stimuli. CM inhibit the mRNA expression of chemokines *CCL2* (**A**), *CCL20* (**B**), *CXCL10* (**E**) and *CXCL11* (**F**) in a dose-dependent way, but have no effect on the mRNA levels of *CXCL1* (**C**) and *CXCL8* (**D**). 2C: IL-1α 5 ng/mL + TNF-α 50 ng/mL; 830 μg/mL total protein of CM; 83 μg/mL total protein of CM. Results are presented as median with interquartile range. **** *p* < 0.0001; *** *p* < 0.001; * *p* < 0.05. N = 3.

**Figure 2:**
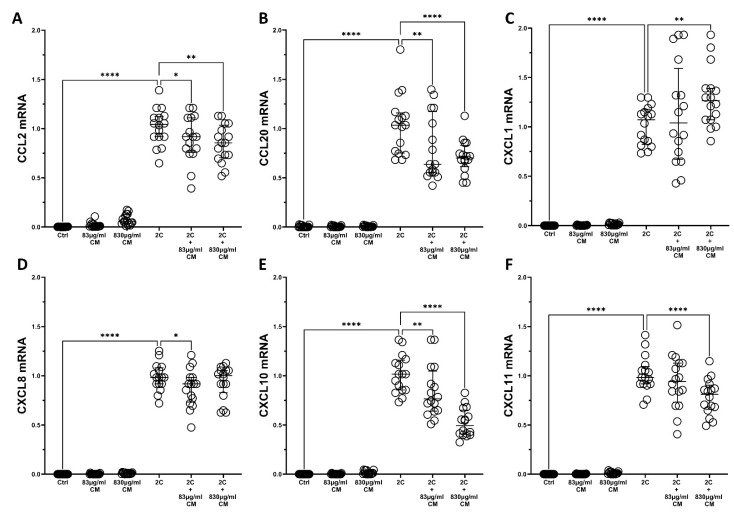
The effect of CM on chemokine mRNA expression in hPSMs, which are under pro-inflammatory stimuli. The CM inhibit the mRNA expression of chemokines *CCL2* (**A**), *CCL20* (**B**), *CXCL10* (**E**) and *CXCL11* (**F**) in a dose-dependent way, but have no effect on the mRNA levels of *CXCL1* (**C**) and *CXCL8* (**D**). 2C: IL-1α 5 ng/mL + TNF-α 50 ng/mL; 830 μg/mL total protein of CM; 83 μg/mL total protein of CM. Results are presented as median with interquartile range. **** *p* < 0.0001; ** *p* < 0.01; * *p* < 0.05. N = 4.

As shown in Figure 1 and Figure 2, IL-1α and TNF-α successfully induced the mRNA expression of the aforementioned chemokines in A549 and hPSMs, and this induction was dose-dependently inhibited, in most cases, by the presence of CM. Specifically, in A549 epithelial cells, the CM inhibited the IL-1α- and TNF-α-inducible mRNA expression of *CCL2* (2C + 830 μg/mL CM: 0.51-fold IQR: 0.48–0.62, *p* < 0.0001; Figure 1A), *CCL20* (2C + 830 μg/mL CM: 0.90-fold IQR: 0.83–0.97, *p* < 0.05; Figure 1B), *CXCL10* (2C + 830 μg/mL CM: 0.30-fold IQR: 0.27–0.67, *p* < 0.0001; Figure 1E) and *CXCL11* (2C + 830 μg/mL CM: 0.37-fold IQR: 0.29–0.72, *p* < 0.0001; Figure 1F) in a dose-dependent way, but had no effect on the mRNA levels of *CXCL1* (Figure 1C) and *CXCL8* (Figure 1D).

The results for hPSMs were similar, where the CM dose-dependently downregulated the mRNA expression of almost all cytokines (Figure 2). With the exception of *CXCL1*, which was found to be statistical significantly upregulated (1.26-fold, IQR: 1.07–1.39, *p* < 0.01; Figure 2C), the CM downregulated the IL-1α- and TNF-α-inducible mRNA expression of *CCL2* (2C + 83 μg/mL CM: 0.91-fold IQR: 0.75–1.11, *p* < 0.05 and 2C + 830 μg/mL CM: 0.85-fold IQR: 0.70–1.03, *p* < 0.01; Figure 2A), *CCL20* (2C + 83 μg/mL CM: 0.63-fold IQR: 0.55–1.17, *p* < 0.01 and 2C + 830 μg/mL CM: 0.70-fold IQR: 0.61–1.82, *p* < 0.0001; Figure 2B), *CXCL8* (2C + 83 μg/mL CM: 0.91-fold IQR: 0.73–0.98, *p* < 0.01; Figure 2D), *CXCL10* (2C + 83 μg/mL CM: 0.76-fold IQR: 0.64–1.04, *p* < 0.01 and 2C + 830 μg/mL CM: 0.49-fold IQR: 0.40–0.64, *p* < 0.0001; Figure 2E), and *CXCL11* (2C + 830 μg/mL CM: 0.81-fold IQR: 0.65–0.90, *p* < 0.0001; Figure 2F).

**Figure 3:**
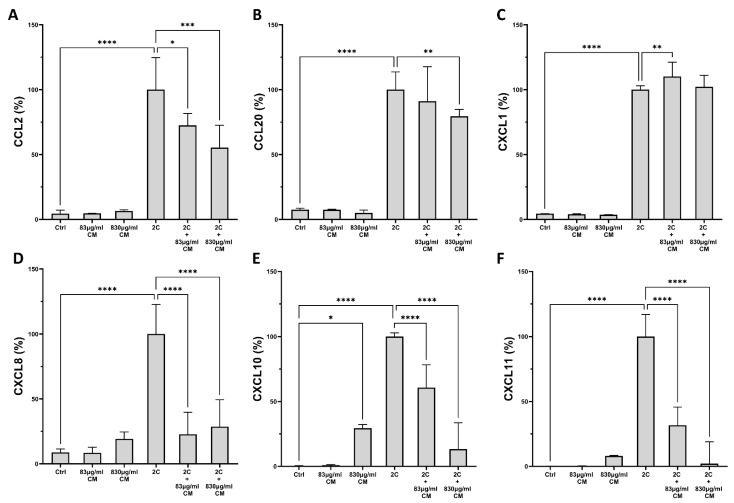
The effect of CM on chemokine protein expression in A549 epithelial cells, which are under pro-inflammatory stimuli. The CM inhibit the protein expression of chemokines CCL2 (**A**), CCL20 (**B**), CXCL8 (**D**), CXCL10 (**E**) and CXCL11 (**F**) in a dose-dependent way, but have no effect on the mRNA levels of CXCL1 (**C**). 2C: IL-1α 5 ng/mL + TNF-α 50 ng/mL; 830 μg/mL total protein of CM; 83 μg/mL total protein of CM. Results are presented as median with interquartile range. **** *p* < 0.0001; *** *p* < 0.001; ** *p* < 0.01; * *p* < 0.05. N = 3.

**Figure 4:**
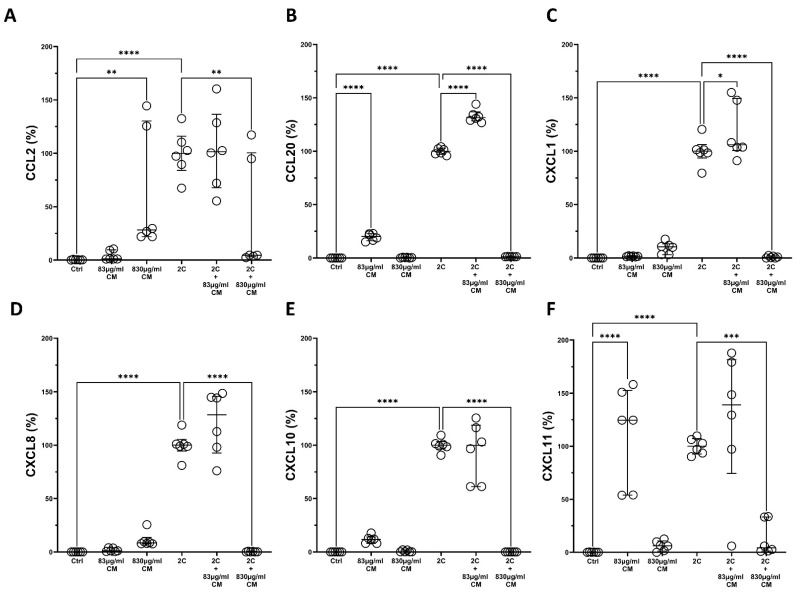
The effect of CM on chemokine protein expression in hPSMs, which are under pro-inflammatory stimuli. The CM inhibit the protein expression of chemokines CCL2 (**A**), CCL20 (**B**), CXCL1 (**C**), CXCL8 (**D**), CXCL10 (**E**) and CXCL11 (**F**) in a dose-dependent way. 2C: IL-1α 5 ng/mL + TNF-α 50 ng/mL; 830 μg/mL total protein of CM; 83 μg/mL total protein of CM. Results are presented as median with interquartile range. **** *p* < 0.0001; *** *p* < 0.001; ** *p* < 0.01; * *p* < 0.05. N = 3.

As shown in Figure 3 and Figure 4, this effect was also confirmed on the protein level for both A549 epithelial cells and hPSMs. Regarding the A549 epithelial cells, the CM had a dose-dependent effect on the IL-1α- and TNF-α-inducible protein expression of CCL2 (2C + 83 μg/mL CM: 72.51% IQR: 61.01–81.67, *p* < 0.05 and 2C + 830 μg/mL CM: 55.28% IQR: 33.19–72.66, *p* < 0.001; Figure 3A), CCL20 (2C + 830 μg/mL CM: 79.51% IQR: 76.81–84.82, *p* < 0.01; Figure 3B), CXCL8 (2C + 83 μg/mL CM: 22.73% IQR: 17.32–39.66, *p* < 0.0001 and 2C + 830 μg/mL CM: 28.67% IQR:15.18–49.37, *p* < 0.0001; Figure 3D), CXCL10 (2C + 83 μg/mL CM: 60.67% IQR: 3.33–78.27, *p* < 0.0001 and 2C + 830 μg/mL CM: 13.39% IQR: 3.70–33.51, *p* < 0.0001; Figure 3E) and CXCL11 (2C + 83 μg/mL CM: 31.70% IQR: 1.84–45.70, *p* < 0.0001 and 2C + 830 μg/mL CM: 2.06% IQR: 0.53–19.05, *p* < 0.0001; Figure 3F), as it statistical significantly downregulated their expressions. Nonetheless, the CXCL1 protein expression remained unaltered by the addition of the higher CM dose and showed a minimal increase when the lower CM dose was present (2C + 83 μg/mL CM: 110.1% IQR: 101.2–121.1, *p* < 0.01; Figure 3C).

Regarding the hPSMs, the CM statistical significantly downregulated the IL-1α- and TNF-α-induced protein production of CCL2 (2C + 830 μg/mL CM: 4.64% IQR: 3.19–100.5, *p* < 0.01; Figure 4A), CCL20 (2C + 830 μg/mL CM: 1.39% IQR: 1.13–1.44, *p* < 0.0001; Figure 4B), CXCL1 (2C + 830 μg/mL CM: 0.70% IQR: 0.01–1.92, *p* < 0.0001; Figure 4C), CXCL8 (2C + 830 μg/mL CM: 0.14% IQR: 0.03–0.58, *p* < 0.0001; Figure 4D), CXCL10 (2C + 830 μg/mL CM: 0.11% IQR: 0.01–0.02, *p* < 0.0001; Figure 4E), and CXCL11 (2C + 830 μg/mL CM: 4.32% IQR: 1.20–33.43, *p* < 0.001; Figure 4F). It should also be noted that, in some cases, either the lower or the higher CM dose statistically significant increased the protein expression of CCL2 (830 μg/mL CM: 28.28% IQR:21.99–130.30, *p* < 0.01; Figure 4A), CCL20 (83 μg/mL CM: 100.00% IQR: 97.16–102.80, *p* < 0.0001; Figure 4B) and CXCL11 (83 μg/mL CM: 124.7% IQR: 54.05–152.8, *p* < 0.0001; Figure 4F), suggesting that it might contain various pro-inflammatory mediators that could induce a mild inflammation of hPSMs.

Taken together, our results indicate that the CM has an indirect anti-inflammatory effect through the downregulation of several chemokines in A549 epithelial cells and hPSMs, ultimately leading to a possible reduced chemotaxis of immune-associated cells.

### 2.2. The Conditioned Medium Regulates the IL-1α- and TNF-α-Inducible mRNA Expression of Several Pro-Inflammatory Interleukins in the Pulmonary Epithelial Cell Line A549 and in hPSMs

Since we found that the CM could downregulate the expression of several chemokines implicated in the chemotaxis of immune-associated cells, we next examined its effect on the IL-1α- and TNF-α-inducible mRNA expression of several pro-inflammatory interleukins in the pulmonary epithelial cell line A549 and in hPSMs. Among the several interleukins, we chose to study the IL-1α, IL-1β, IL-4, IL-6, IL-10, IL-13, IL-17, IL-22 and TNF-α, as they play major roles in the Th1, Th2 and Th17 immune responses [63].

**Figure 5:**
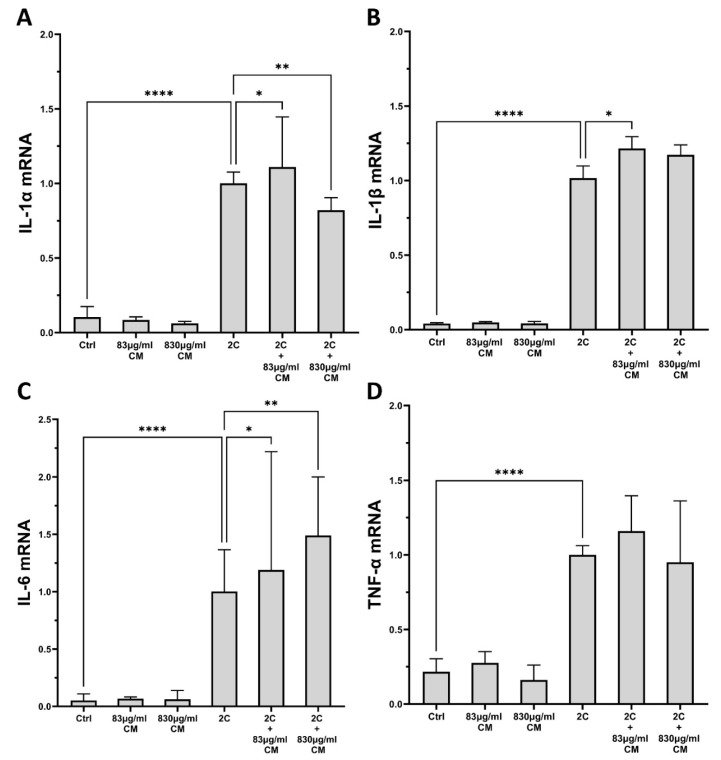
The effect of CM on interleukin mRNA expression in A549 epithelial cells, which are under pro-inflammatory stimuli. A higher CM dose downregulates the mRNA expression of *IL-1α* (**A**), and a lower CM dose upregulates the mRNA expression of *IL-1β* (**B**), either CM dose increases the mRNA expression of *IL-6* in a dose-dependent way (**C**) and has no effect on the mRNA expression levels of *TNF-α* (**D**). 2C: IL-1α 5 ng/mL + TNF-α 50 ng/mL; 830 μg/mL total protein of CM; 83 μg/mL total protein of CM. Results are presented as median with interquartile range. **** *p* < 0.0001; ** *p* < 0.01; * *p* < 0.05. N = 3.

As shown in Figure 5, the IL-1α and TNF-α combination statical significantly upregulated the pro-inflammatory interleukins, *IL-1α*, *IL-1β*, *IL-6* and *TNF-α*, in A549 cells, compared to controls. The addition of either CM dose alone had no effect on these interleukin expressions, while its presence under the IL-1α and TNF-α pro-inflammatory stimuli had various effects. Of the four interleukins, the higher CM dose statistical significantly downregulated the IL-1α- and TNF-α-inducible mRNA expression of *IL-1α* (2C + 830 μg/mL CM: 0.82-fold IQR: 0.68–0.90, *p* < 0.01; Figure 5A), while its lower dose induced a mild, but statistically significant, increase (2C + 83 μg/mL CM: 1.11-fold IQR: 1.01–1.44, *p* < 0.05; Figure 5A). Regarding *IL-1β*, a statistically significant increase was observed in its mRNA expression when A549 cells were stimulated with both pro-inflammatory cytokines and the lower CM dose (2C + 83 μg/mL CM: 1.19-fold IQR: 1.07–1.41, *p* < 0.05; Figure 5B), and the results for the *IL-6* mRNA expression, where a dose-dependent increase was seen when the CM doses were added, were similar (2C + 83 μg/mL CM: 1.19-fold IQR: 0.93–1.73, *p* < 0.05 and 2C + 830 μg/mL CM: 1.49-fold IQR: 1.31–1.63, *p* < 0.01; Figure 5C). Regarding *TNF-α*, although we observed an increase in its mRNA expression by the presence of either CM dose and the combination of IL-1α and TNF-α, no increase was statistically significant (Figure 5D). All of the other studied interleukins were found undetectable in A549 epithelial cells (data not shown).

**Figure 6:**
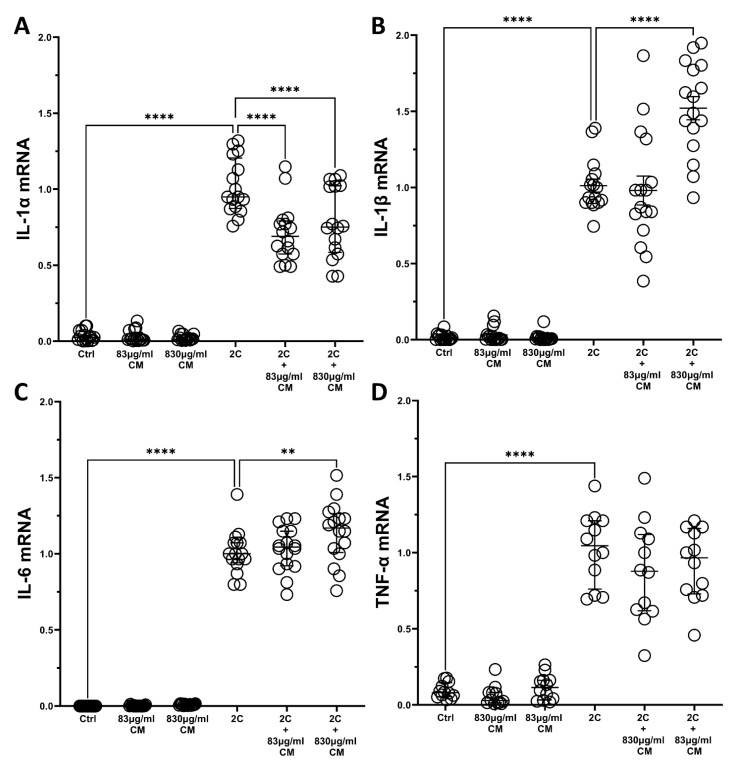
The effect of CM on interleukin mRNA expression in hPSMs, which are under pro-inflammatory stimuli. Both CM doses downregulate the mRNA expression of *IL-1α* (**A**), the higher CM dose upregulates the mRNA expression of *IL-1β* (**B**) and *IL-6* (**C**), and no effect on the mRNA expression levels of *TNF-α* (**D**) is observed. 2C: IL-1α 5ng/mL + TNF-α 50ng/mL; 830 μg/mL total protein of CM; 83 μg/mL total protein of CM. Results are presented as median with interquartile range. **** *p* < 0.0001; ** *p* < 0.01. N = 4.

The results on hPSMs were similar, where the IL-1α and TNF-α combination statistical significantly upregulated the pro-inflammatory interleukins *IL-1α*, *IL-1β*, *IL-6* and *TNF-α*, compared to controls, while the addition of either CM dose alone had no effect on these interleukin expressions (Figure 6). When hPSMs were stimulated with both the IL-1α and TNF-α combination and either CM dose, the *IL-1α* mRNA expression was statistical significantly downregulated (2C + 83 μg/mL CM: 0.68-fold IQR: 0.57–0.79, *p* < 0.0001 and 2C + 830 μg/mL CM: 0.75-fold IQR: 0.58–1.02, *p* < 0.0001; Figure 6A), while the IL-1α- and TNF-α-inducible mRNA expression of *IL-1β* was statistically significant increased by the addition of the higher CM dose (2C + 830 μg/mL CM: 1.54-fold IQR: 1.30–1.79, *p* < 0.0001; Figure 6B). Regarding *IL-6*, the higher CM dose statistical significantly upregulated the IL-1α- and TNF-α-inducible mRNA expression of *IL-6* (2C + 830 μg/mL CM: 1.16-fold IQR: 1.00–1.26, *p* < 0.01; Figure 6C), but neither CM dose had an effect on the IL-1α- and TNF-α-inducible mRNA expression of *TNF-α* (Figure 6D). Again, the rest of the studied interleukins were also found to be undetectable in hPSMs (data not shown).

Altogether, our results indicate that the CM may downregulate the expression of the pro-inflammatory IL-1α, but they could also further induce a pro-inflammatory response in both the A549 epithelial cells and the hPSMs.

### 2.3. The Effect of the Conditioned Medium on the TGF-β-Induced Fibrotic Responses of hPSMs

Having confirmed that the CM have an anti-inflammatory effect on hPSMs, we next investigated its effect on the TGF-β-induced fibrotic responses of hPSMs, and, in particular, the mRNA expression of *collagen Type I*, *Type III*, and *fibronectin* and the protein expression of the total secreted collagen.

**Figure 7:**
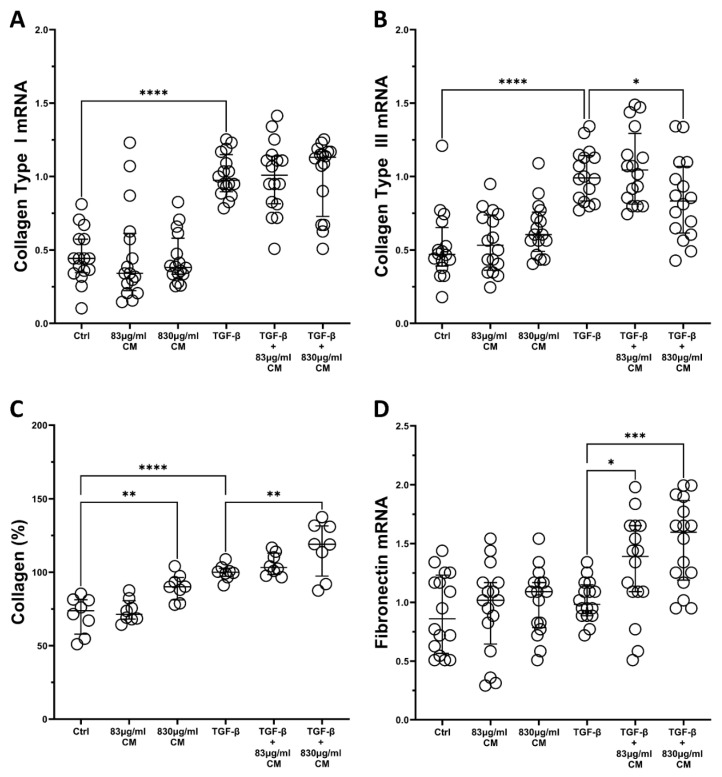
The effect of CM on fibrotic expression in hPSMs, which are under pro-fibrotic stimuli. Both CM doses do not alter the TGF-β-inducible mRNA expression of *Collagen Type I* (**A**), the higher CM dose downregulates the mRNA expression of *collagen Type III* (**B**), but upregulates the protein production of total secreted collagen (**C**), and both CM doses upregulate the TGF-β-inducible mRNA expression of *fibronectin* (**D**). TGF-β: 5 ng/mL; 830 μg/mL total protein of CM; 83 μg/mL total protein of CM. Results are presented as median with interquartile range. **** *p* < 0.0001; *** *p* < 0.001; ** *p* < 0.01; * *p* < 0.05. N = 4.

Regarding *collagen Type I*, TGF-β statistical significantly upregulated its mRNA levels but the addition of either CM dose did not alter this inducible increase (Figure 7A). On the other hand, the higher CM dose had a statistically significant effect on the TGF-β-inducible mRNA levels of *collagen Type III*, as it downregulated it (2C + 830 μg/mL CM: 0.83-fold IQR: 0.61–1.07, *p* < 0.05; Figure 7B). Nonetheless, when it came to measuring the protein production of the total secreted collagen, we found that the CM alone statistical significantly increased the total protein collage secretion, compared to control (830 μg/mL CM: 90.14% IQR: 81.18–96.43, *p* < 0.01; Figure 7C), and it also statistical significantly increased its TGF-β-inducible protein levels in a dose-dependent way (2C + 830 μg/mL CM: 119.1% IQR: 97.42–131.6, *p* < 0.01; Figure 7C).

Finally, regarding the *fibronectin* expression, although TGF-β did not induce its mRNA expression, both CM doses statistical significantly increased its expression only when TGF-β was also present (2C + 83 μg/mL CM: 1.39-fold IQR: 1.09–1.65, *p* < 0.05 and 2C + 830 μg/mL CM: 1.59-fold IQR: 1.19–1.86, *p* < 0.001; Figure 7D).

### 2.4. The Conditioned Medium Inhibits the TGF-β-Induced Migration of hPSMs

**Figure 8:**
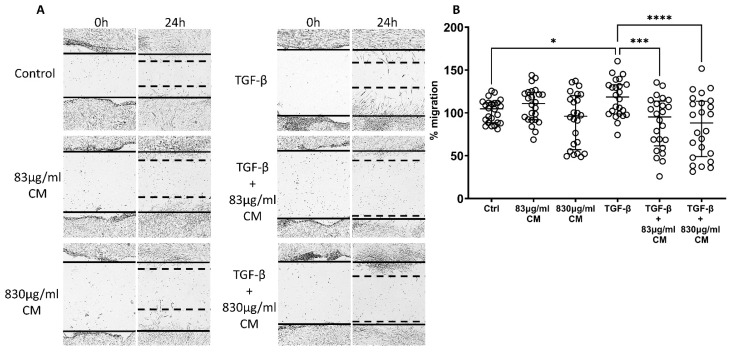
The effect of CM on the migration capacity of hPSMs, which are under pro-fibrotic stimuli. Both CM doses were statistically significant in decreasing the TGF-β-inducible migration of hPSMs (**A**,**B**). TGF-β: 5ng/mL; 830 μg/mL total protein of CM; 83 μg/mL total protein of CM. Results are presented as median with interquartile range. **** *p* < 0.0001; *** *p* < 0.001; * *p* < 0.05. N = 4.

Next, we examined the possible effect of CM on the TGF-β-induced migration of hPSMs. As shown in Figure 8B, TGF-β statistical significantly increased the migration rate of hPSMs (118.60% IQR: 98.81–132.9, *p* < 0.05;) compared to untreated cells, while the CM abolished this TGF-β-induced increase in migration rate in a dose-dependent way. Specifically, the CM were statistically significant in decreasing the TGF-β-induced migration rate (TGF-β + 83 μg/mL CM: 95.28% IQR: 61.48–113.80, *p* < 0.001 and TGF-β + 830 μg/mL CM: 88.28% IQR: 49.04–113.60, *p* < 0.0001; Figure 8B), suggesting that the CM could have a therapeutic effect on fibrosis, as they could halt the TGF-β-induced migration rate of hPSMs.

## 3. Discussion

In this study, we showed that the CM derived from an immortalized human-adipose-derived mesenchymal stem cell line have a clear anti-inflammatory effect as they inhibited the mRNA and protein expression of chemokines in A549 cells and hPSMs in a dose-dependent way. They also downregulated the mRNA levels of *IL-1α* in both of these cell types. We also observed that the CM had a mild and indirect anti-fibrotic effect on hPSMs, as they could downregulate the mRNA expression levels of *collagen Type III* and could decrease the TGF-β-inducible migration rate. These results suggest that the CM could have a direct effect on chemotaxis and cell recruitment, including leukocytes and myofibroblasts, and indirectly, an anti-inflammatory effect. This chemotactic and cell recruitment effect, in a second step, could affect fibrosis, given that cell recruitment, apart from inflammation, is also involved in the fibrotic process.

More specifically, a higher CM dose downregulated the IL-1α- and TNF-α-inducible mRNA expression of several chemokines in both A549 cells and hPSMs, and this mRNA downregulation was also translated to extremely decreased protein levels. We chose to investigate the effect of CM on the IL-1α- and TNF-α-inducible CCL and CXCL expression, because both of these two chemokine families exerted their pro-inflammatory actions through the recruitment of immune-associated cells, and it was also shown that CXCL chemokines played a role in the migration of fibroblasts and endothelial cells [64]. Regarding IPF, various chemokines were found to be increased or were associated with disease exacerbation. In particular, the CCL2 chemokine was found to be increased in bronchoalveolar lavage fluid (BALF) [65] and activated myofibroblasts of patients with IPF [66], suggesting that this chemokine has a major role in both the recruitment of immune-associated cells and in the process of fibrogenesis. In addition, CCL2, together with CXCL10, have been characterized as prognostic biomarkers for the disease progression and the survival outcome of patients with IPF [67], and in a murine model of chronic obstructive pulmonary disease the chemokines CCL2, CXCL1, CXCL8, CXCL10 and CXCL11 were found to be elevated and correlated with the establishment of fibrosis [68]. All of this evidence suggests that chemokines may play a double role in IPF, recruiting immune-associated cells and fibroblasts, and thus prolonging inflammation and promoting fibrogenesis.

Our results agree with other studies showing that mesenchymal stem cells play a positive role in regulating immune responses and attenuating pulmonary fibrosis. Cargnomi et al. showed that human amniotic MSCs could promote the polarization of T regulatory cells, and anti-inflammatory M2 macrophages and could also decrease the recruitment and maturation of B immune cells in a murine model of pulmonary fibrosis [69]. In another study of radiation-induced lung injuries, which utilized MSCs modified with decorin, a natural inhibitor of TGF-β, treatment with these modified MSCs resulted in reduced lymphocyte infiltration, decreased expression levels of pro-inflammatory chemokines and cytokines, and inhibition of fibrogenesis, but also increased the expression of anti-inflammatory cytokines [70]. Recently, we also showed that the CM could ameliorate pulmonary fibrosis and inflammation in an in vivo model, through the reduced expression of pro-inflammatory cytokines and the decreased recruitment of immune-associated cells [48].

Regarding the CM effect on the combined IL-1α- and TNF-α-inducible mRNA expression of interleukins, we observed that the CM downregulated the *IL-1α*, but upregulated the *IL-1β* and *IL-6*. In previous studies, IL-1α was found to be elevated in a bleomycin-induced fibrosis model [71], and autoantibodies to this cytokine were also found in the serum of patients with rapidly progressive IPF [72], suggesting that IL-1α could play a significant role in the progression of IPF, and thus treatment with CM could contribute to its downregulation. Regarding IL-1β, other studies using MSCs as treatment for pulmonary inflammation and fibrosis showed a decrease in its expression [72,73], but this difference in our results could be attributed to the fact that we used CM and not the MSCs themselves. As mentioned above, we observed an upregulation of the IL-1α- and TNF-α-inducible mRNA expression of *IL-6* in both A549 cells and hPSMs when treated with CM. Various other studies and our study showed that MSCs or the CM from MSCs could ameliorate fibrosis and inflammation in vivo, and in particular, they could downregulate the expression of IL-6 [37,42,48,74,75,76]. Again, this disagreement with our results could be attributed to the fact that this study is an in vitro study, examining the CM effect on simple cell cultures and not on the possible different cellular interactions that can be seen in in vivo studies.

As far as the anti-fibrotic effect of the CM is concerned, we observed that the CM had a mild and indirect effect on fibrosis, as it was statistically significant in downregulating the TGF-β-inducible mRNA levels of *collagen Type III* and the migration rate of hPSMs, but it also upregulated the mRNA levels of *fibronectin* and the total protein collagen production. Previous studies from other researchers and our laboratory have shown that the CM indeed have an anti-fibrotic effect on pulmonary fibrosis. Li et al. showed that the treatment with bone marrow mesenchymal stem cell (BMSC)-derived CM in rats with silica-induced pulmonary fibrosis resulted in a reduced overall collagen deposition, along with decreased mRNA levels of *collagen I*, *collagen III*, and fibronectin, TGF-β1 and hydroxyproline [77]. Utilizing the same animal model, Zhao et al. reported that the administration of BMSCs led to a decreased collagen deposition and hydroxyproline content, and it also downregulated the mRNA levels of *collagen Type I* and *fibronectin* [78]. Apart from the in vivo data, BMSC-derived CM were found to inhibit the in vitro epithelial-to-mesenchymal transition. Wang et al. observed that the A549 epithelial cells treated with CM had reduced levels of the mesenchymal-associated markers α-SMA and vimentin and increased levels of the epithelial markers, E-cad and CK8 [79]. Similar results were reported utilizing other types of MSCs. We showed that ADSC-CM ameliorated pulmonary fibrosis in Bleomycin-treated mice, as it reduced the collagen deposition in their lungs [48]. Felix et al. showed that the administration of both ADSCs and their CM in Bleomycin-treated rats resulted in a significant overall improvement, with reduced levels of TGF-β and collagen I fibers [43]. All of these studies lead to the conclusion that MSCs and their CM indeed have an anti-fibrotic effect on pulmonary fibrosis, and although in this study we found increased protein levels of the total collagen production, the migration rate of hPSMs was reduced in a statistically significant manner. Therefore, one could speculate that the CM could exert their anti-fibrotic actions by decreasing the migratory capacity of hPSMs, and if the most major fibrogenic cell, the myofibroblast, could not migrate, fibrogenesis could become stalled.

Possible failure in discovering an all-encompassing single compound pharmacological drug for the successful treatment of IPF may be due to the limitation of a single mechanism of action in what seems to be quite a multimodal disease mechanism. Regenerative biologicals, such as CM, because of their multifactorial constituents, have multiple mechanism of action, as depicted in this study, with both an anti-inflammatory and anti-fibrotic effect that may be plausible additions to the treatment regime for such diseases.

## 4. Materials and Methods

### 4.1. Adipose-Derived Mesenchymal Stem Cell Isolation and Immortalization

Adipose-derived mesenchymal stem cells were isolated and immortalized, as previously described [48]. Briefly, subcutaneous adipose tissue from a 35-year-old healthy female, who was informed and agreed to participate in this study by giving her written consent, was obtained during outpatient tumescence liposuction under local anesthesia. All principles outlined in the Declaration of Helsinki for all human experimental investigations were followed. Next, the adipose-derived stem cells (ADSCs) were immortalized as previously described [48], by transducing with the human telomerase reverse transcriptase (hTERT) gene in combination with lentiviral gene SV-40. ADSCs’ phenotype was verified by confirming the expression of CD90 and CD105 and the lack of expression of Cd34 under a fluorescent microscope (Leica DM2000, Leica Microsystems GmbH, Wetzlar, Germany), as shown in Supplementary Figure S1A.

### 4.2. Characterization of hPSMs and ADSCs

The hPSMs and ADSCs were characterized by immunofluorescence as previously described [80]. Briefly, cells were first fixed in 4% paraformaldehyde (PFA; Sigma-Aldrich, St. Louis, MO, USA) for 40 min at 4 °C, then membrane permeabilization was achieved by a 15 min incubation with 0.1% Triton-X (Sigma-Aldrich, St. Louis, MO, USA) and blocking was performed using 5% bovine serum albumin (BSA; Sigma-Aldrich, St. Louis, MO, USA) for 1 h. Primary antibodies (Novus Biologicals, Littleton, CO, USA) against the markers of interest were added and left overnight at 4 °C, and secondary fluorochrome-conjugated antibodies (Novus Biologicals, Littleton, CO, USA) were added the following day and left for 2 h. Finally, nuclei were stained with DAPI (Sigma-Aldrich, St. Louis, MO, USA) and samples were observed under a fluorescent microscope (Leica DM2000, Leica Microsystems GmbH, Germany).

### 4.3. Conditioned Medium Preparation

Conditioned media (CM) from ADSCs were collected and processed as previously described [48]. A total of 1 × 10^5^ cells were plated in T-75 flasks in medium containing DMEM (PeproTech EC, Ltd., London, UK), 10% FBS (Sigma-Aldrich, St. Louis, MO, USA) and gentamycin 1% (Biosera, Nuaillé, France), at 37 °C and 5% CO_2_. Upon the cells becoming confluent, the media were changed to the same media, excluding FBS. Supernatants were collected every 72 h and fresh FBS-free medium was added. Cell supernatants were harvested and centrifuged at 2500 rpm for 10 min at 4 °C and filtered through a 0.45-micron filter (Merck-Millipore, Burlington, MA, USA) to remove cell debris and cryopreserved at −80 °C until use. Total protein of CM was calculated to be 830 μg/mL using Quawell Q5000 UV-Vis Spectometer (Quawell, San Jose, CA, USA).

### 4.4. Patients

Healthy pulmonary tissue, with no histopathological evidence of disease, was obtained from patients that underwent lobectomy for primary pulmonary tumor removal. The local Research Ethics Committee of the University Hospital of Alexandroupolis approved this study, and all patients gave their informed written consent prior to participation.

### 4.5. Isolation of Human Pulmonary Subepithelial Myofibroblast

Human pulmonary subepithelial myofibroblasts (hPSMs) were isolated from surgical specimens of healthy lung tissue, as previously described [81]. Briefly, tissue specimens were collected in ice-cold Hank’s balanced salt solution (HBSS; Biosera, Nuaillé, France) with Ca/Mg plus penicillin, streptomycin, amphotericin B, and gentamicin (Biosera, Nuaillé, France) and were de-epithelialized using 1 mM dithiothreitol (DTT; Sigma-Aldrich, St. Louis, MO, USA), and then with 3 mM ethylene-diaminetetraacetic acid (EDTA; Sigma-Aldrich, St. Louis, MO, USA). Epithelial-denuded tissue was finally incubated in Dulbecco’s Modified Eagle Medium (DMEM, PeproTech EC, Ltd., London, UK) plus 10% fetal bovine serum (FBS; Sigma-Aldrich, St. Louis, MO, USA) in 5% CO_2_ at 37 °C. During culturing, numerous non-adherent and adherent cells appeared in the culture flasks. Cells in suspension were removed every 72 h. Denuded tissue was maintained in culture for up to 4 weeks, until numerous foci of myofibroblasts appeared attached to the bottom of the flasks. Tissue specimens were then removed, and hPSMs were cultured in DMEM (PeproTech EC, Ltd., London, UK) supplemented with 10% FBS (Sigma-Aldrich, St. Louis, MO, USA). The myofibroblast phenotype was verified by confirming the expression of a smooth muscle actin (a-SMA) and vimentin and the lack of expression of desmin in a fluorescent microscope (Leica DM2000, Leica Microsystems GmbH, Wetzlar, Germany), as shown in Supplementary Figure S1B.

### 4.6. A549 Pulmonary Epithelial Cell Line

A549 is a cell line of alveolar basal epithelial cells that were isolated from a pulmonary adenocarcinoma of a 58-year-old Caucasian male [82]. A549 cells were cultured in DMEM (PeproTech EC, Ltd., London, UK) supplemented with FBS (Sigma-Aldrich, St. Louis, MO, USA) and antibiotics.

### 4.7. Stimulation of A549 and hPSMs with Recombinant Cytokines and Conditioned Medium

hPSMs at passages 2–5 were used in our studies. All experiments were performed with FBS-free media at 95% culture confluence with a stable ratio of supernatant volume-to-surface available for cell adhesion (1.5 mL:9.6 cm^2^).

hPSMs and A549 cells were either untreated or treated for 6 or 48 h with: (a) 830 μg/mL CM, (b) 83 μg/mL CM, (c) a combination of 5 ng/mL IL-1α (R&D Systems, Minneapolis, MN, USA) and 50 ng/mL TNF-α (R&D Systems, Minneapolis, MN, USA), (d) a combination of 5 ng/mL IL-1α and 50 ng/mL TNF-α and 830 μg/mL CM, (e) a combination of 5 ng/mL IL-1α and 50 ng/mL TNF-α and 83 μg/mL CM, (f) 5 ng/mL TGF-β (R&D Systems, Minneapolis, MN, USA), (g) 5 ng/mL TGF-β and 830 μg/mL CM or (h) 5 ng/mL TGF-β and 83 μg/mL CM. At the end of the 6 h incubation period, hPSMs and A549 cells were lysed with Nucleozol Nucleozol (MACHEREY-NAGEL, Düren, Germany) and kept at −80 °C until assayed. At the end of the 48 h incubation period, supernatants and total cells were collected and kept at −80 °C until assayed.

### 4.8. Total RNA Extraction and DNase Treatment

Total RNA from hPSMs and A549 cells were extracted using Nucleozol (MACHEREY-NAGEL, Düren, Germany) according to the manufacturer’s instructions and as previously described [48]. In short, H_2_O was added to each sample, following incubation and a 15 min centrifugation at 12.000 g. Supernatants were then incubated with isopropanol and centrifuged for 10 min at 12.000 g in order to precipitate the RNA. The RNA pellet was washed twice with 75% ethanol and reconstituted with H_2_O. Total RNA concentration was measured using Quawell Q5000 UV-Vis Spectometer (Quawell, San Jose, CA, USA). Any DNA contaminations were removed using Recombinant DNase I (TaKaRa, Kusatsu, Shiga, Japan).

### 4.9. cDNA Synthesis and Real-Time PCR

Next, 200 ng of the DNAse treated RNA was reverse transcripted using the PrimeScript 1st strand cDNA Synthesis Kit (TaKaRa, Kusatsu, Shiga, Japan) according to the manufacturer’s instructions. Following cDNA synthesis, 25 ng cDNA was then amplified using qRT-PCR Sybr Green (Kapa Biosystems, Wilmington, NC, USA) and 100 nΜ of gene-specific primers, shown in Table 1, in SaCycler-96 RUO (Sacace Biotechnologies, Como, Italy). A two-step amplification protocol was performed for all studied genes and the gene expression of each studied gene was normalized against GAPDH gene expression in the same sample using the 2-ΔΔCt method.

### 4.10. Enzyme Linked Immunosorbent Assay (ELISA)

Human DuoSet^®^ ELISAs (R&D Systems, Minneapolis, MN, USA) were used to estimate the protein concentrations of CCL2, CCL10, CXCL1, CXCL8, CXCL10 and CXCL11 chemokines in hPSMs and A549 cells’ supernatants, according to the manufacturer’s instructions and as previously described [61]. Briefly, flat 96-well plates were coated overnight with capture antibody for each chemokine, and the following day, plates were incubated with the recommended blocking buffer for 2 h. Next, duplicates of each supernatant and known concentrations of chemokine samples were added in wells, incubated for 2 h, and then, biotinylated detection antibody for each chemokine was added for another 2 h. Streptavidin-horseradish peroxidase was then added for 20 min and the following addition of tetramethylbenzidine with H_2_O_2_ produced different optical densities (OD) of color which were measured at 450 nm on a microplate reader (Diareader EL×800; Dialab, Wr. Neudorf, Austria). The chemokines concentration was calculated using a linear standard curve according to the manufacturer’s instructions.

### 4.11. Measurement of Total Protein Collagen Production

Collagen was measured with the Sircol assay (Sircol; Biocolor, Carrickfergus, UK), as previously described [80]. Briefly, ice-cold Isolation & Concentration Reagent was added to hPSMs’ supernatants, and samples were incubated overnight at 4 °C. The next day, samples were centrifuged at 13,000× *g* for 10 min, and Sirius Red Dye was then added. After a 30 min incubation, samples were centrifuged at 13,000× *g* for 10 min, supernatant was discarded, and the collagen pellet was dissolved in 0.5 M NaOH alkali reagent. Next, the optical densities (ODs) of the samples and controls of known collagen concentration were measured at 540 nm in a microplate reader (Diareader ELx800; Dialab, Wr. Neudorf, Austria). The collagen concentration was calculated using a linear standard curve according to the manufacturer’s instructions.

### 4.12. Wound Healing Assay

The effect of CM on the TGF-β-induced migration of hPSMs was assessed in vitro with the wound-healing scratch assay, as previously described [80]. A narrow gap devoid of cells was created on confluent hPSMs cultures, and next, we measured the rate of gap closure. This process resembles wound healing and is dependent on myofibroblast migration, hyperplasia, and proliferation. hPSMs were cultured in 6-well plates, and when confluence was reached, 1 mechanical wound per well was performed with a 10-μL pipette tip. The wound was vertical to predrawn lines on the bottom of the well so that we could define 3 stable points per well at the junctions of those lines with the wound. Images were recorded at those fixed wound points with an inverted Olympus microscope equipped with a camera. hPSMs were treated with various cytokines (as previously described) for 24 h, and images were taken at time points 0 and 24 h. The average percentage of gap closure after 24 h in treated wells was divided by the average percentage of gap closure in untreated wells to assess the treatment effect.

### 4.13. Statistics

Statistical analyses were performed using Prism Software 9 (GraphPad Software, San Diego, CA, USA). Results are presented as median with interquartile range (IQR). Comparison of values among sample groups was performed with ordinary one-way ANOVA. Statistical significance was set at *p* < 0.05.

## 5. Conclusions

In conclusion, we showed that ADSC-derived CM could directly affect the chemotaxis and the cell recruitment of immune-associated cells, as they strongly reduced the expression of various chemokines, and in a second phase, they could also indirectly affect fibrogenesis, as the migration capacity of hPSMs was decreased, and cell recruitment was implicated in the fibrosis process. More studies are needed to further elucidate the anti-inflammatory and anti-fibrotic mechanisms of the CM and to possibly identify the responsible soluble mediators, through which the CM exerts its therapeutic actions.

## Figures and Tables

**Table 1:** Gene-specific primer sequences used in our study.

Gene	Forward	Reverse	Reference
GapdH	GACATCAAGAAGGTGGTGAA	TGTCATACCAGGAAATGAGC	[80]
COL1	CCCTGGAAAGAATGGAGATGAT	ACTGAAACCTCTGTGTCCCTTCA	
COL3	GCTCTGCTTCATCCCACTATTA	TGCGAGTCCTCCTACTGCTAC	
FD-A	CCAGTCCACAGCTATTCCTG	ACAACCACGGATGAGCTG	
CCL2	AGGAAGATCTCAGTGCAGAGG	AGTCTTCGGAGTTTGGGTTTG	[83]
CCL20	GCTGCTTTGATGTCAGTGC	GCAGTCAAAGTTGCTTGCTTC	[84]
CXCL1	GCCCAAACCGAAGTCATAGCC	ATCCGCCAGCCTCTATCACA	[85]
CXCL8	TGGGTGCAGAGGGTTGTG	CAGACTAGGGTTGCCAGATTTA	[83]
CXCL10	CCTGCTTCAAATATTTCCCT	CCTTCCTGTATGTGTTTGGA	
CXCL11	GACGCTGTCTTTGCATAGGC	GGATTTAGGCATCGTTGTCCTTT	[86]
IL-1α	AGATGCCTGAGATACCCAAAACC	CCAAGCACACCCAGTAGTCT	[87]
IL-1β	ACAGATGAAGTGCTCCTTCCA	GTCGGAGATTCGTAGCTGGAT	[88]
IL-4	ACTTTGAACAGCCTCACAGAG	TTGGAGGCAGCAAAGATGTC	[89]
IL-6	AAGCCAGAGCTGTGCAGATGAGTA	TGTCCTGCAGCCACTGGTTC	[90]
IL-10	CATCAAGGCGCATGTGAACT	GATGTCAAACTCACTCATGGCTTT	[91]
IL-13	TGAGGAGCTGGTCAACATCA	CAGGTTGATGCTCCATACCAT	[89]
IL-17	GTCAACCTGAACATCCATAACCG	ACTTTGCCTCCCAGATCACAG	[92]
IL-22	TTCCAGCAGCCCTATATCACC	GCTCACTCATACTGACTCCGTG	[93]
TNF-α	CCCAGGGACCTCTCTCTAATC	ATGGGCTACAGGCTTGTCACT	[88]

## Data Availability

The data presented in this study are available on request from the corresponding author.

## Supplementary Materials

The following supporting information can be downloaded at: https://www.mdpi.com/article/10.3390/ijms23094570/s1.
