# Supplementary material for: Anti-Inflammatory and Anti-Fibrotic Effect of Immortalized Mesenchymal-Stem-Cell-Derived Conditioned Medium on Human Lung Myofibroblasts and Epithelial Cells
Source: Int J Mol Sci. 2022 Apr 20;23(9):4570. doi: 10.3390/ijms23094570 (PMC9102072; doi:10.3390/ijms23094570)
Supplement: Supplementary file 1 [file ijms-23-04570-s001.zip › ijms-1681828-supplementary.pdf]

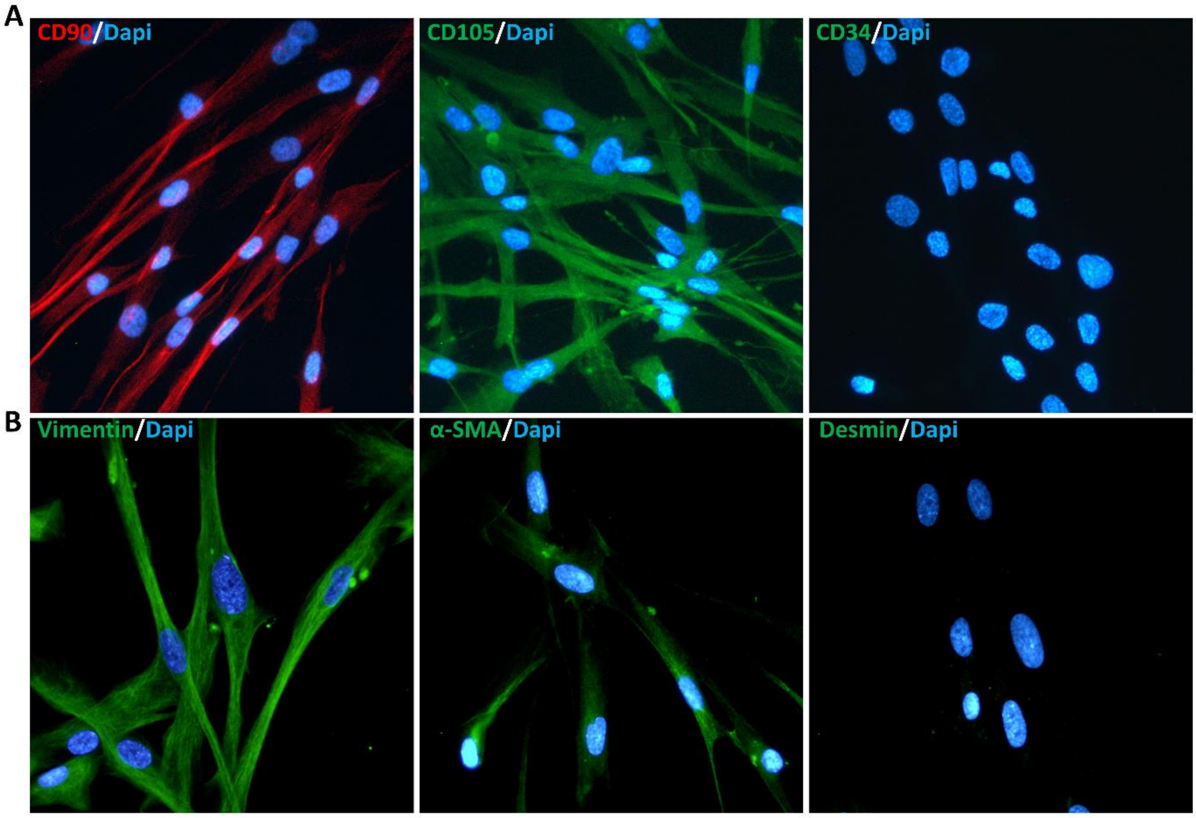

Figure S1. Characterization of ADSCs and hPSMs. (**A**) ADSCs were found to be positive for CD90 and CD105 and negative for CD34. (**B**) hPSMs were found to be positive for vimentin and α-SMA and negative for desmin. Representative 400× immunofluorescence snapshots are shown. Blue and red/green colors correspond to DAPI and antibody staining, respectively.
